# *Camelina neglecta* (Brassicaceae, Camelineae), a new diploid species from Europe

**DOI:** 10.3897/phytokeys.115.31704

**Published:** 2019-01-17

**Authors:** Jordan R. Brock, Terezie Mandáková, Martin A. Lysak, Ihsan A. Al-Shehbaz

**Affiliations:** 1 Department of Biology, Washington University in St. Louis, St. Louis, MO 63130, USA Washington University St. Louis United States of America; 2 CEITEC – Central European Institute of Technology, Masaryk University, Kamenice 5, 625 00 Brno, Czech Republic Masaryk University Brno Czech Republic; 3 Missouri Botanical Garden, 4344 Shaw Boulevard, St. Louis, Missouri 63110, USA Missouri Botanical Garden St. Louis United States of America

**Keywords:** Brassicaceae, *
Camelina
*, Camelineae, chromosome numbers, Cruciferae

## Abstract

*Camelina neglecta* is described as a new diploid species and its relationship to the other diploids of the genus and to the somewhat superficially similar tetraploid *C. rumelica* and hexaploid *C. microcarpa*, are discussed. SEM of seed and stem trichomes of the new species are presented.

## Introduction

The Brassicaceae (Cruciferae) is an economically important family with ca. 4050 species and 348 genera (BrassiBase 2018, Kiefer et al. 2014, Koch et al. 2018, DA German and MA Koch pers. com.). It includes many crops such as broccoli, Brussels sprouts, cabbage, cauliflower, canola, turnip (*Brassica* L.), radish (*Raphanus sativus* L.), arugula (Eruca vesicaria subsp. sativa (Mill.) Thell.), horseradish (*Amoracia rusticana* Gaetnr., Mey., & Scherb.), wasabi (*Eutrema japonicum* (Miq.) Koidz.) and watercress (*Nasturtium officinale* W.T.Aiton), as well as *Arabidopsis thaliana* (L.) Heynh., the model organism in modern biology.

*Camelina* Crantz, a small genus of seven or eight Eurasian species, has become increasingly interesting due to ongoing research in developing *C. sativa* (L.) Crantz as a high-yielding crop for oilseed and aviation biofuel. Wild populations of *Camelina* species may harbour agronomically important traits for introgression and crop improvement and attention to these has heightened in recent decades. Several *Camelina* species occur as cosmopolitan weeds (*C. sativa*, *C. microcarpa* Andrz. and *C. rumelica* Velen.), whereas others have restricted ranges in the Irano-Turanian floristic region, predominantly Turkey.

One of the authors (JRB) studied the *Camelina* accessions in the United States Department of Agriculture’s (USDA) Germplasm Resource Information Network collection and, based on flow cytometry, he noticed that accession 650135 had a small genome size, consistent with diploidy. Both Galasso et al. (2015) and Martin et al. (2017) showed that plants of that accession are diploid with 2*n* = 12, whereas Martin et al. (2018) found the existence of sexual incompatibility between plants of that accession and the morphologically similar hexaploid *C. microcarpa*. In light of these findings and based on a critical evaluation of morphology of plants of *C. microcarpa* and *C. rumelica*, we recognise plants of that accession as the following new species.

## Taxonomy

### Camelina neglecta

Taxon classificationPlantaeBrassicalesBrassicaceae

J.Brock, Mandáková, Lysak & Al-Shehbaz sp. nov.

8643E5B0EC7F50FA87337BEEC970A763

urn:lsid:ipni.org:names:77193889-1

Figs 1 , 2 , 3 , 4

#### Type.

France, Lozere, Causse Méjean, corn field, September 1996, 44°16'N, 2°33'E, *Henri Besancon s.n.* (holotype: MO-6869197; isotype: MO-6869196).

#### Description.

Annual herbs. Stems 50–60 cm tall, simple at base, branched about middle or above, densely pilose above base with exclusively simple, crisped trichomes 1–3 mm long, glabrous at middle and above. Basal leaves withered by anthesis; cauline leaves oblong-lanceolate, middle ones 4–5.5 × 0.5–1 cm, gradually reduced in size upwards and becoming narrowly lanceolate, sparsely hirsute with simple trichomes, ciliate with antrorse subsetose trichomes 0.1–1 mm long, base sagittate, margin entire, apex acute. Racemes 30–75-flowered, becoming lax, elongated considerably and 18–24 cm long in fruit; fruiting pedicels 0.9–2 cm long, divaricate-ascending, glabrous. Sepals oblong, 2–2.5 mm long; petals pale yellow, narrowly oblanceolate, 2.5–4.5 × 0.8–1 mm; median filaments ca. 2 mm long; anthers ovate, ca. 0.2 mm long; ovules 30–34(–36) per ovary. Fruit pyriform, 7–7.5 × 4– 4.5 mm; valves not veined, margin strongly carinate, winged, apex acuminate, extending 0.9–1.1 mm on to stylar area; style 1.3–1.6 mm long, free portion only ca. 0.5 mm long. Seeds brown, oblong, 0.9–1.1 × 0.5–0.6 mm; seed coat minutely papillate, copiously mucilaginous when wetted.

**Figure 1.**
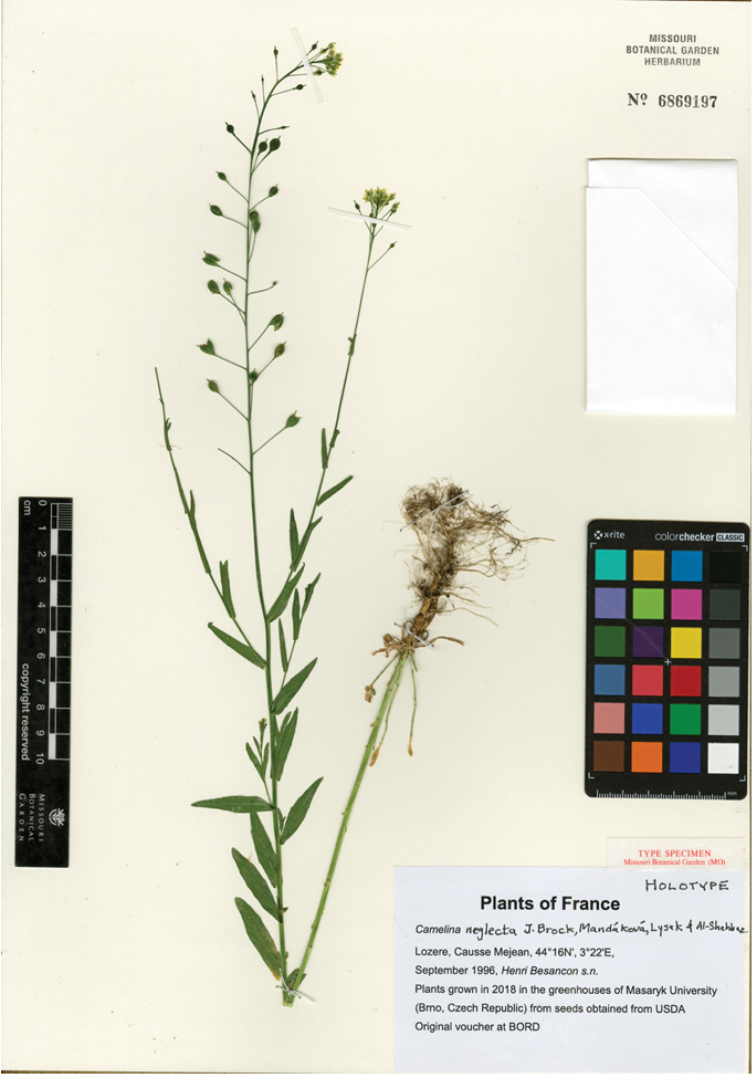
Holotype of *Camelina neglecta*. *Besancon s.n.* (MO-6869197).

The origin of the type material is a seed collection deposited at the USDA and no original voucher is known anywhere, including BORD, long suspected to house it. As a result, a greenhouse-grown plant from the USDA seeds was pressed as the voucher and therefore is recognised as the holotype.

**Figure 2.**
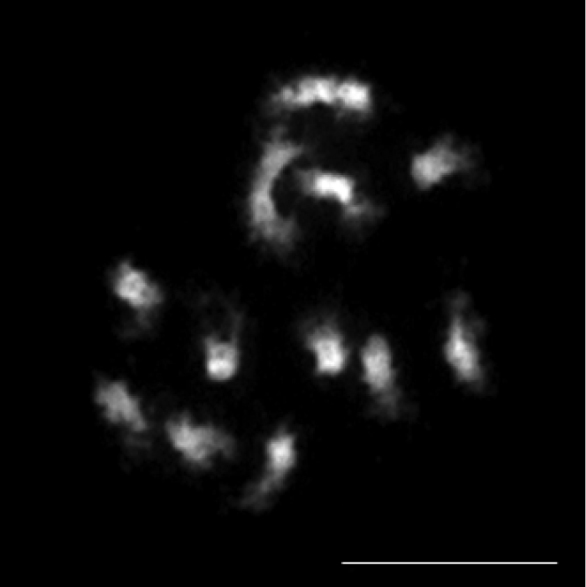
Mitotic chromosomes of *Camelina neglecta*. Greenhouse-grown plants from seeds of *Besancon s.n.* (USDA accession 650135). Scale bar: 10 μm.

*Camelina neglecta* is a diploid species most closely resembling the hexaploid (2*n* = 40) *C. microcarpa* DC. and the tetraploid (2*n* = 26) *C. rumelica*. Deviant counts for *C. microcarpa* are almost certainly based on misidentifications of plants of other species. For example, counts of 2*n* = 26 for *C. microcarpa* from France, Morocco and Spain (see Warwick and Al-Shehbaz 2006, BrassiBase) most likely belong to *C. rumelica*, a species two of the authors (MAL and TM) found to consistently have 2*n* = 26. Furthermore, diploid (2*n* = 12) counts for *C. rumelica*, from Hungary (Baksay 1957) and United States (Brooks 1985), are most likely based on plants of *C. neglecta* or another diploid species yet to be described. Critical verifications of the vouchers of these previous counts are needed to establish their identity beyond any doubt. One of the authors (IAS) examined the voucher cited in Brooks (erroneously reported as McGregor 35289 instead of 35290; Freeman, pers. com.) and it fits quite well in *C. neglecta*, based on trichome morphology and ovule number. Our count of 2*n* = 12 in *C. neglecta* (Fig. 2) agrees with this and is based on the same seed accession as that of Martin et al. (2017), misidentified as *C. microcarpa*. The present isolated occurrence of *C. neglecta* in France might appear to be odd, but with the availability of resources, a thorough search for it in eastern Europe and Southwest Asia should be made.

**Figure 3.**
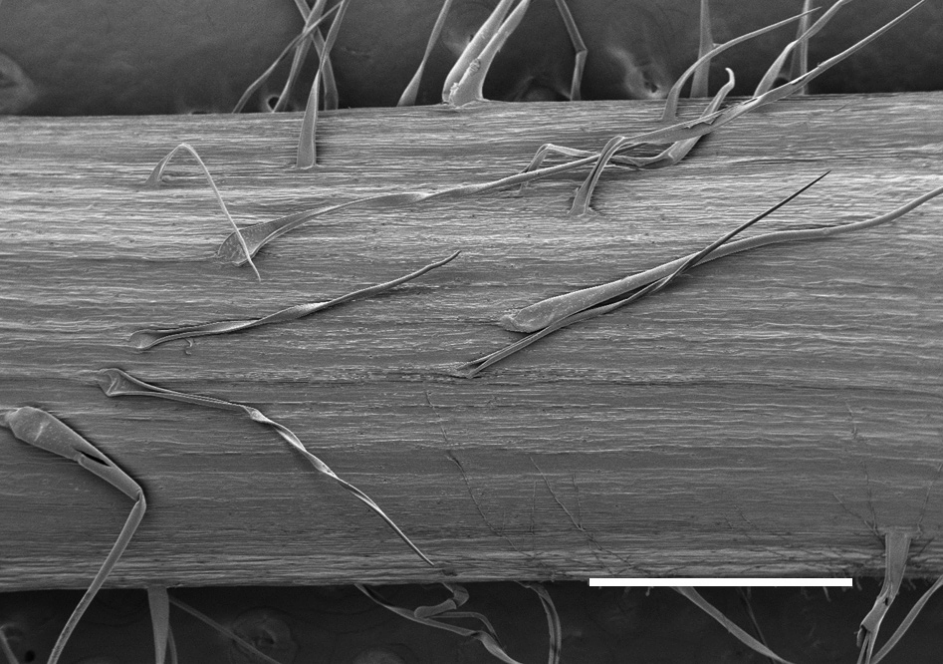
Trichomes of lowermost part of stem in *Camelina neglecta*. Greenhouse-grown plants from seeds of *Besancon s.n.* (USDA accession 650135). Scale bar: 400 μm.

In addition to differences in ploidy level and chromosome numbers, *Camelina neglecta* differs from both *C. microcarpa* and *C. rumelica* by having lower stems soft pilose (vs. hirsute) with crisped (vs. straight) trichomes not mixed (vs. mixed) with forked ones (Fig. 3), as well as by having 30–34(–36) [vs. (16–)20–24(–26)] ovules per ovary. It further differs from the yellow-flowered *C. microcarpa* by having petals 2.5–4.5 (vs. 3.8–6) mm long petals and fruit 7– 7.5 (vs. 4–5.5) mm long. From *C. rumelica, C. neglecta* also differs by the smaller yellow (vs. white) petals 2.5–4.5 (vs. (5–)6–9) mm long and pilose (vs. hirsute) lower stems.

There are two other Southwest Asian diploid species in the genus, of which *Camelina laxa* C.A.Mey. (2*n* = 12) is distributed in Armenia, Azerbaijan, Georgia, Iran and Turkey and it is unique in the genus in having strongly flexuous infructescences. The other is *C. hispida* Boiss. (2*n* = 14), a species of Iran, Iraq, Israel, Jordan, Lebanon, Syria and Turkey. The latter differs from all other species of the genus by having pubescent (vs. glabrous) middle stems and inflorescences.

**Figure 4.**
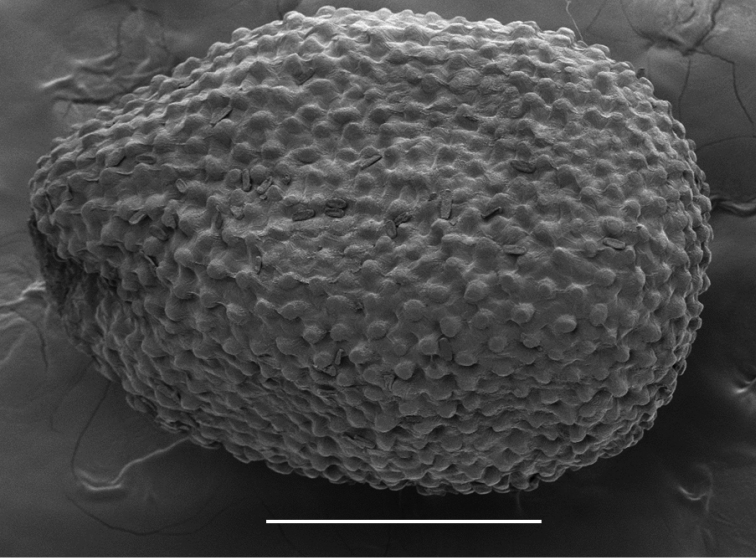
SEM image of *Camelina neglecta* seed. Greenhouse-grown plants from seeds of *Besancon s.n.* (USDA accession 650135). Scale bar: 1 mm.

The papillate seeds of *Camelina neglecta* (Fig. 4) are copiously mucilaginous and the seed epidermis exudes the mucilage within a few seconds after soaking.

The native ranges of five *Camelina* species (*C. hispida, C. laxa, C. microcarpa, C. rumelica* and *C. sativa*) are widespread in south-eastern Europe and/or Southwest Asia (especially Turkey). Other species, *C. anomala* Boiss. & Hausskn. and *C. stiefelhagenii* Bornm., are rare in Turkey but appeared in areas outside of their known native range, with a collection of *C. anomala* from Beqaa, Lebanon (1961) and *C. stiefelhagenii* from Dresden, Germany (1941) and Gothenburg, Sweden (1952). It is quite possible that *C. neglecta* is more widespread in Europe and SW Asia that we currently know.

Due to the allohexaploid nature of *Camelina sativa*, there is much interest in discovering its putative diploid parents. The phylogenetic treatment of the genus (Brock et al. 2018) showed the relationships of diploid *Camelina* species relative to *C. sativa* and indicated a potentially shared hybridisation and polyploidisation history of the weedy *C. microcarpa* and its domesticated *C. sativa*. It is essential to identify the wild *Camelina* diploids to facilitate reconstruction of the evolutionary history of *C. sativa* and allow the potential for re-synthesis of the crop as has been done in *Brassica napus* L. (Chen et al. 1988).

## Supplementary Material

XML Treatment for Camelina neglecta

## References

[B1] Baksay L (1957) The chromosome numbers and cytotaxonomical relations of some European plant species. Annales historico-naturales Musei Nationalis Hungarici, Budapest 8: 169–174.

[B2] BrassiBase (2018) https://brassibase.cos.uni-heidelberg.de/ [Accessed 4 November 2018]

[B3] Brock JR Dönmez AA Beilstein MA Olsen KM (2018) Phylogenetics of *Camelina* Crantz. (*Brassicaceae*) and insights on the origin of gold-of-pleasure (*Camelina sativa*). Molecular Phylogenetics and Evolution 127: 834–842. 10.1016/j.ympev.2018.06.031 29933039

[B4] Brooks RE (1985) Chromosome number reports LXXXVII. Taxon 34: 347.

[B5] Chen BY Heneen WK Jönsson R (1988) Resynthesis of *Brassica napus* L. through Interspecific Hybridization between *B. alboglabra* Bailey and *B. campestris* L. with Special Emphasis on Seed Colour. Plant Breeding 101(1): 52–59. 10.1111/j.1439-0523.1988.tb00266.x

[B6] Galasso I Manca A Braglia L Ponzoni E Breviario D (2015) Genomic fingerprinting of *Camelina* species using cTBP as molecular marker. American Journal of Plant Sciences 6(08): 1184–1200. 10.4236/ajps.2015.68122

[B7] Kiefer M Schmickl R German DA Mandáková T Lysak MA Al-Shehbaz IA Franzke A Mummenhoff K Stamatakis A Koch MA (2014) BrassiBase: Introduction to a novel knowledge database on Brassicaceae evolution. Plant Cell Physiology 55(1): e3(1‒9). 10.1093/pcp/pct158 24259684

[B8] Koch MA German DA Kiefer M Franzke A (2018) Database taxonomics as key to modern plant biology. Trends in Plant Science 23(1): 4–6. 10.1016/j.tplants.2017.10.005 29146431

[B9] Martin SL Smith TW James T Shalabi F Kron P Sauder CA (2017) An update to the Canadian range, abundance, and ploidy of *Camelina* spp. (Brassicaceae) east of the Rocky Mountains. Botany 95(4): 405–417. 10.1139/cjb-2016-0070

[B10] Martin SL Lujan‐Toro BE Sauder CA James T Ohadi S Hall LM (2018) Hybridization rate and hybrid fitness for *Camelina microcarpa* Andrz. ex DC (♀) and *Camelina sativa* (L.) Crantz (Brassicaceae) (♂). Evolutionary Applications (Early View). 10.1111/eva.12724 PMC6383699 30828366

[B11] Warwick SI Al-Shehbaz IA (2006) Brassicaceae: Chromosome number index and database on CD-Rom. Plant Systematics and Evolution 259(2–4): 237–248. 10.1007/s00606-006-0421-1

